# The Use of Mallein for the Diagnosis of Glanders in Horses and Experiments with an Albumose Extracted from Cultures of the Bacillus Malleus

**Published:** 1892-11

**Authors:** E. A. De Schweinitz, F. L. Kilborne

**Affiliations:** Chemist, Bureau of Animal Industry, Department of Agriculture, Washington, D. C.; Director of Veterinary Experiment Station, Bureau of Animal Industry, Department of Agriculture, Washington, D. C.


### THE JOURNAL
OF
### COMPARATIVE MEDICINE AND VETERINARY ARCHIVES.

VOL. XIII. NOVEMBER, 1892. No. 11.

### THE USE OF MALLEIN FOR THE DIAGNOSIS OF GLANDERS IN HORSES AND EXPERIMENTS WITH AN ALBUMOSE EXTRACTED FROM CULTURES OF THE BACILLUS MALLEUS.

BY

E. A. DE SCHWEINITZ, PH.D., *Chemist*, F. L. KILBORNE, B.V.S., *Director of Veterinary Experiment Station*,

*Bureau of Animal Industry, Department of Agriculture, Washington, D. C.*

In December, 1890, we extracted from culture liquids of the bacillus malleus an albumose which appeared to be the active principle in these cultures. At that time a preliminary experiment was conducted to see if this substance could be used to make guinea pigs immune to the disease of glanders. The result was that out of a set of five, three vaccinated and two checks, only one, a vaccinated animal, recovered from an inoculation of a glanders culture. This experiment has since been repeated on sets of ten and twelve guinea pigs each, with, at the present writing, only negative results. A note of this work was published in the annual report of the Department of Agriculture for 1891. The albumose was best obtained from the cultures, after the removal of the germ by means of a Pasteur filter, by precipitation with absolute alcohol, resolution in water and reprecipitation.

In the July number, 1892, of the Archives de Medecines Experimentales et d'anatomie Patholigique, A. Babes describes the isolation in the same way of apparently the same substance from glanders cultures. He claims with this to have been able to immunize guinea pigs against glanders. He has either failed to see or note our earlier experiments.

During the present year we have, at the direction of Dr. D. E. Salmon, turned our attention to the use of mallein as a means of diagnosing glanders.

In the experiments made by Preusse, Nocard,[1] Tietze,[2] Eber,[3] and others, the mallein used was prepared by scraping the surface growth from potato cultures, dissolving in water, sterilizing by heating to 100 degrees C., filtering and injecting upon the addition of an equal volume of a two per cent. solution of carbolic acid.

Our mallein was prepared in the following way: Acid peptonized beef broth cultures, containing 5 per cent. of glycerine, were inoculated with the glanders bacillus and the cultures allowed to grow for two months at the temperature of the room. At this time the growth, which had been very abundant, had almost entirely ceased. The liquid was then heated for two hours from 80 to 100 degrees C., after which it was filtered through a Pasteur tube to remove the germs. The resulting clear amber liquid, after being tested to prove the absence of the germs, and diluted with 50 per cent. of glycerine for better preservation, was used for injections. A purer lymph was obtained by the precipitation of the filtered cultures with absolute alcohol and resolution in 50 per cent. glycerine. For practical purposes the crude lymph is equally good. The greatest care and precautions have to be observed in all of the work, that the cultures are pure, of the proper age, and that the lymph is perfectly sterile, and can be so preserved. Care must also be observed in making the injection to avoid the danger of contamination or infection by means of the syringe.

The value of the mallein prepared as described, as a diagnostic agent for glanders in horses, has been extensively tried at the experiment station of the Bureau of Animal industry; and also by Drs. Francis of Texas, Dinwiddie of Arkansas, and Caswell of Chicago. Lymph was also sent to several other experiment stations and veterinarians, but they have as yet failed to report. The results of these tests are recorded in the accompanying tables.

As sent out for use, the lymph is diluted with an equal volume of glycerine so that it will keep better.

The amounts of mallein named in the charts refer to the undiluted solution. Of the diluted solution just double the quantity was given.

At first we made injections with 1cc. of the mallein (2cc. of

1. Nocard Recueil de Med. Veter. 1892, p. 209.
3. Eber " " " " " p. 86.
2. Tietze Berl. Thierarz. Woch. 1892, No. 8, p. 86.

the 50 per cent. solution), but finding this to be apparently too large a dose, as it sometimes gave a decided febrile reaction in healthy horses, we have decreased the quantity to 0.5cc. of the mallein, or 1cc. of the 50 per cent. solution. But judging from the results being obtained from some experiments now in progress, we may find it desirable to retnrn to the larger dose and depend on a second injection of those horses which give a doubtful reaction for a positive diagnosis.

In several instances the same horses have been repeatedly injected. To indicate this in the charts, a figure in brackets is placed at the head of the column showing the temperatures, which figure indicates the number of times the horse has been injected.

With the exception of a few of the earlier injections which were made over the shoulder, we have made the injection near the middle of one side of the neck, where any local swelling can be readily detected. In our experiments the local reaction or swelling in the glandered horse, has been more constant and fully as characteristic as the febrile reaction.

The following notes refer to the tests made with mallein as recorded in Table I. The numbers correspond to the numbers of the horses. The notes were recorded by the veterinarians making the tests.

Horse No. 1. Horse was 7 or 8 years old, the right submaxilliary gland was enlarged, there was a discharge from the right nostril, but no farcy buds. The condition of the animal was good.

The day after the injection there was an increased flow from the right nostril and a painful enlargement at the point of injection.

No. 2. Horse was 6 to 7 years old. Both submaxillary glands were enlarged, and there was a slight nasal discharge. The condition of the animal was good. It had been affected for six to seven months.

No. 3. This animal was four years old, both submaxillary glands were enlarged, but there was no nasal discharge. Right hand leg swollen. There were thirteen farcy buds. The general condition was poor.

No. 4. The condition of the animal was fair and there were farcy buds on front legs and on the breast. The respiration was labored. There was no nasal discharge, the submaxillary glands were not enlarged, but the left eye was mattering. Dr. Francis diagnosed a probable mild pneumonia in addition to the farcy.

No. 5. Horse was about nine years old. Both submaxillary glands were enlarged, there was some nasal discharge. Condition of the animal was very bad, farcy buds and chancres on both hind legs, veins of forelegs enlarged. The animal urinated frequently.

No. 6. This animal was perfectly healthy.

No. 7. This animal was six years old, in good flesh. She had been exposed to glanders about five weeks before injection. She was suckling a foal. The foal was kept from her for twelve hours previous to taking the temperature before injection. After the injection and during the test the foal was returned to her.

No. 8. This animal was a two-year-old filly, diseased with glanders, in good flesh and apparently feeling well. The owner insisted upon having her shot immediately after the temperature was taken the last time.

No. 9. This animal was an aged mare which had been exposed to glanders five weeks before the injection. She was in very good flesh and condition.

No. 10. This was a three-year-old entire horse, small size, well nourished, healthy. A painful swelling formed at point of injection in four hours, and persisted forty-eight hours. Maximum temperature was reached ten hours after injection, and was 1.7 degrees F. above that at the same time the previous day.

No. 11. This was a four-year-old gelding in fair condition, healthy, weight 1050 pounds. The local swelling persisted for two days, and temperature rose to 0.7 degrees F. above that at the same time the previous day.

No. 12. This animal was an old mare with chronic glanders, both lung and nasal lesions. Irregular temperature, usually above normal. Large local swelling for two days. Maximum temperature was reached twelve hours after the injection, and was 2.2 degrees F. above that at the corresponding time the day before.

No. 12. Reinjected. Maximum temperature was reached ten hours after making the injection, and was 2.5 degrees F. above that at the corresponding time the day before. The local swelling persisted for three days.

As for the notes on the horses which were injected by this Bureau, all of which were kept very carefully, it is not necessary to go into detail here. In general we may say that the glandered horses, except No. 14, were pronounced cases of the disease, having been condemned for glanders and farcy, in the District of Columbia, and sent to the veterinary station to be destroyed. Nos. 7, 23, 25, 31, 34, 35, and 39 were cases of glanders with well-marked nasal lesions. Nos. 10, 19, 20, 21, and 26 showed only symptoms of farcy (although No. 19 developed nasal lesions after the injections.) The only symptoms shown by No. 14 before injection were an elevated temperature, 101 to 102.5 degrees F., and an enlarged, rounded (not nodular), left submaxillary gland, with a history of coming from a stable from which two horses were removed and condemned for glanders Sept. 21, 1891. From this date until Feb. 13, 1892, the mare continued in excellent condition and the best of spirits, with no perceptible change in the above symptoms, although freely purged with aloes two or three times during the period. Two days after the first injection there appeared a slight nasal discharge, followed by an attack of acute glanders, the animal dying March 26.

Guinea pigs were inoculated from farcy buds or with the nasal discharge from horses numbered 7, 20, 23, 25, and 26, the pigs so inoculated all contracting glanders.

The healthy horses used, Nos. 8, 9, 11, 12, 13, 15, 16, 17, 18, 22, 24, 27, 29, 30, 33, 36, 37, and 38 were in general in good condition at the time of the experiment.

The glandered horses, except those with a high temperature at the time of the injection (Nos. 34, 35 and 39), all showed a marked rise in temperature in from four to eight hours after the first injection, and usually reached the maximum in from ten to sixteen hours after the injection. Further, there was in all cases a characteristic very tender abrupt swelling at the point of the first injection and also at all subsequent injections except in Nos. 19, 20 (9th injection) and 34, generally beginning to appear two to four hours after the injection, continuing on the next day and increasing in size for from one to three days, disappearing again three to nine days after the injection.

Some of the healthy horses showed a slight rise of temperature (No. 8 a marked elevation) upon the first injection, but only in one instance, horse No. 38, did this rise persist on the second injection. This latter horse has unfortunately been lost sight of, and sold. It was to all appearances free from disease when hired for the injection, but the rise of temperature upon two successive injections would look suspicious, although it did not show the characteristic swelling of glanders after either injection. In the healthy horses in no instance was there very marked swelling at the seat of the injection of doses not exceeding 1 c.c. of the mallein. When it did appear it developed in from two to six hours after the injection, and had disappeared again by the next day.

Horse No. 7 received a daily injection of 1 c.c. of the mallein from May 11 to June 23, inclusive. During this time there was no elevation of temperature, which varied from 99.5° to 101.6° F. But after each injection a local swelling appeared, varying in extent from six to ten inches across, and disappearing at the end of about the third day. During the last week of the injections the swellings were less marked, but still invariably followed each injection. Otherwise no effect was noted from the continued treatment.

On horse No. 21 the farcy buds had entirely healed within a week after the first injection, and on No. 20 the extensive farcy swellings had all healed July 15th. Except a slight discharge from the left ear of No. 20, these two animals have continued apparently entirely well and have improved in general health since the above dates. However, the last injections of the mallein, August 27 and 30, caused a decided rise in temperature, and a marked characteristic swelling at the point of injection, which would indicate that the horses are not free from disease.

Table V shows the effect upon horses, of the albumose extracted from the cultures. In the first of these experiments the amount used, 0.005 grams, was evidently entirely too large, causing almost as much reaction in the healthy as in the glandered horses. In the second and third experiments the dose was reduced to 0.002 grams per horse, but this was also slightly too large. This table serves to show the powerful effect of the poison secreted by the glanders bacillus. A dose of only 0.001 grams of the albumose would probably be sufficient to give a decided reaction.

In conclusion, the tests made show that the mallein is of great value in the diagnosis of glanders in horses. The injection of 0.5 or 1 c.c. of the mallein may cause a slight rise of temperature in a healthy horse, but it will very rarely give a reaction upon a second injection ; and there will not occur on the healthy horse any marked swelling at the seat of the injection. On the glandered horse the first injection always gives a marked febrile reaction (except as noted) and a large swelling at the seat of the injection. Subsequent injections also give a similar reaction, although frequently less marked. Healthy horses readily acquire an immunity to large doses of the mallein, not so acquired by glandered horses.

The active principle in mallein is an albumose which can be precipitated from the cultures by means of alcohol or ammonium sulphate.

Further experiments may prove that a preventive injection or treatment of glanders in horses is possible.

The directions which were sent out by the Bureau of Animal

Industry to serve as a guide in testing the value of the mallein may be conveniently given here for future reference:

Make the test, if possible, with a healthy horse as well as with one or more affected with glanders. Take the temperature of all these animals three times a day for one or two days before making the injection. On the day of making the injection, take the temperature every two hours from early in the morning until late in the evening. Use for each horse one cubic centimeter of the solution as sent you, and make the injection beneath the skin of the shoulder. Be careful to thoroughly sterilize the syringe after injecting each horse, or better, use separate syringes for healthy and suspected animals. If the same syringe must be used, inject the healthy animals first, to thoroughly sterilize the syringe after each of the other injections. Sterilize the thermometer in carbolic acid after taking the temperature of each horse. The temperature will begin to rise, as a rule, from three to four hours after the injection, and reach its maximum eight to ten hours after injection. On the two days succeeding the injection, take the temperature three times a day. Note the general condition of the animal both before and after the injection. After four or five days the injection should be repeated.

Keep the solution in a sealed bottle, and in a cool place. As sent from here, it is free from germs.

So far as we have experimented the mallein has caused a rise in the temperature of all horses affected with glanders; but it is possible that it also, in rare instances, causes a rise in the temperature of horses that are not affected with this disease. We deem it very important that this latter point should be definitely determined by a considerable number of observations.

*See following pages for Tables of Experiments.*

### TABLE No. I.

### *Experiments with 1 cc. Mallein.*

Tests by M. Francis, College Station, Tex.

| | Date. | Time. | Gland'd. No. 1. Temp. | Gland'd. No. 2. Temp. | Gland'd. No. 3. Temp. | Gland'd. No. 4. Temp. | Gland'd. No. 5. Temp. |
|---|---|---|---|---|---|---|---|
| Before Injection. | July 19, | 9 a m | 100. | 99.9 | 100.6 | 102. | 101.6 |
| | " | 2 p m | 100. | 100.6 | 101.6 | 102.2 | 101.8 |
| | " | 5.30 p m | 100.6 | 100.7 | 101.6 | 102.2 | 101.8 |
| | July 20, | 9 a m | 100. | 99.9 | 100.9 | 104.4 | 101.4 |
| | " | 2 p m | 100.4 | 100.6 | 101.9 | 102.9 | 101.8 |
| | | | Injected at 7 a m. | | Injected at 7 a m. | | |
| When Injected. | July 21, | 9 a m | 100. | 100. | 100. | 102.5 | 101.4 |
| | " | 11 a m | 102.4 | 102. | 101.8 | 102.9 | 102.3 |
| | " | 2 p m | 103.5 | 102. | 103.5 | 103.5 | 102.7 |
| | " | 5 p m | 104.2 | 103.4 | 103.9 | 103.5 | 102.8 |
| | " | 6.30 p m | 103.5 | 104. | 104.2 | 103.1 | 103.4 |
| | | | Swelling at point of injection. | Swell'g. | Swell'g. | Swell'g. | Swell'g. |
| After Injection. | July 22, | 10 a m | 103. | 101.9 | 101.5 | 103.8 | 102.3 |
| | " | 2 p m | 103.2 | 102. | 102.4 | 102. | 103.8 |
| | " | 5.30 p m | 102.2 | 101.5 | 102. | 101.5 | 102.2 |

Tests by John Caswell, Chicago, Ill.

| Date. | Healthy. No. 6. Time. | Temp. | Date. | Healthy. No. 7. Time. | Temp. | Glandered. No. 8. Time. | Temp. | Healthy. No. 9. Time. | Temp. |
|---|---|---|---|---|---|---|---|---|---|
| July 24, | 9 a m | 99.8 | July 12, | 8 a m | 104.4 | 8 a m | 102.4 | 8 a m | 100. |
| " | 4 p m | 100.2 | " | 11 a m | 100.4 | 12 m | 100.4 | 11 a m | 100.2 |
| " | 6 p m | 100.2 | " | 5.30 p m | 100.6 | 6 p m | 101.2 | 5.30 p m | 100.4 |
| | Injected at 7 a m. | | | | | | | | |
| July 25, | 8.30 a m | 99.8 | July 13, | 8.30 a m | 100. | 8.30 a m | 100.8 | 8.30 a m | 100. |
| " | 10.30 a m | 100.2 | " | 10.30 a m | 100.2 | 10 30 a m | 101. | 10.30 a m | 100.2 |
| " | 12.30 p m | 100.1 | " | 12.30 p m | 100.8 | 12.30 p m | 102.4 | 12.30 p m | 101.4 |
| " | 2.30 p m | 101. | " | 3 p m | 100.8 | 3.20 p m | 104.8 | 3 p m | 100.6 |
| " | 4.30 p m | 101.4 | " | 6 p m | 100.4 | 5 p m | 104.8 | 6 p m | 100.4 |
| " | 6.30 p m | 101.8 | | | | 6 p m | 104.8 | | |
| " | 8.30 p m | 101. | | | | | | | |
| | No Swelling. | | | | | | | | |
| July 26, | 6.30 a m | 99.8 | July 14, | 8.30 a m | 100.2 | 8.30 a m | 105.4 | 8.30 a m | 99.8 |
| " | 8.30 a m | 100. | " | 3 p m | 100. | 3 p m | 103.4 | 3 p m | 99.8 |
| " | 10.30 a m | 100.8 | " | 5.30 p m | 100. | 5.30 p m | 102. | 5.30 p m | 99.8 |
| " | 12.30 p m | 102. | | | | | | | |
| " | 2.30 p m | 100. | | | | | | | |
| " | 4.30 p m | 100. | | | | | | | |
| " | 6.30 p m | 100. | | | | | | | |

Tests by R. R. Dinwiddie, Fayetteville, Ark.

| Healthy. | | | Healthy. | | | Glandered. | | | Reinjected (2). | | |
|---|---|---|---|---|---|---|---|---|---|---|---|
| No. 10. | | | No. 11 | | | No. 12. | | | No. 12. | | |
| Date. | Time. | Temp. | Date. | Time. | Temp. | Date. | Time. | Temp. | Date. | Time. | Temp |
| Ap. 4, | 9 a m | 99.6 | Ap. 8, | 9.30 a m | 99.2 | Ap. 12, | 6.30 a m | 99.9 | Ap. 18, | 9 a m | 101. |
| " | 3 p m | 100.8 | " | 2 p m | 100. | " | 8.30 a m | 99.7 | " | 3 p m | 101. |
| " | 7 p m | 100.1 | " | 6.30 p m | 100 2 | " | 10.30 a m | 100. | " | 7 p m | 101.3 |
| Ap. 5, | 6.20 a m | 99.8 | Ap. 9, | 6 a m | 100. | " | 12.30 p m | 100.9 | | | |
| " | 8.20 a m | 99. | " | 10 a m | 99.9 | " | 3.30 p m | 100.9 | | | |
| " | 10.20 a m | 99. | " | 2.30 p m | 99.2 | " | 4.30 p m | 101. | | | |
| " | 12.20 p m | 99.5 | " | 8 p m | 100.6 | " | 6.30 p m | 101.7 | | | |
| " | 2.20 p m | 100. | " | 10 p m | 100.4 | " | 8.30 p m | 101. | | | |
| " | 4.20 p m | 100.3 | | | | " | 10.30 p m | 101. | | | |
| " | 6.20 p m | 100.5 | | | | | | | | | |
| " | 8.20 p m | 100.3 | | | | | | | | | |
| Injected at 6.20 a m. | | | Injected at 6.45 a m. | | | Injected at 6.30 a m. | | | Injeeted at 7 a m. | | |
| Ap. 6, | 6.20 a m | 99.2 | Ap. 10, | 6.45 a m | 99.3 | Ap. 13, | 6.30 a m | 100.3 | Ap. 19, | 7 a m | 100.4 |
| " | 8.20 a m | 99.2 | " | 8.45 a m | 98.6 | " | 8.30 a m | 100.5 | " | 9 a m | 100.8 |
| " | 10.20 a m | 99.4 | " | 10.45 a m | 100. | " | 10.30 a m | 101.3 | " | 11 a m | 101.7 |
| " | 12.20 p m | 100. | " | 12.45 p m | 99. | " | 12.30 p m | 101.9 | " | 1 p m | 101.9 |
| " | 2.20 p m | 101.3 | " | 2.45 p m | 100.3 | " | 2.30 p m | 102.9 | " | 3 p m | 102.7 |
| " | 4.20 p m | 102. | " | 4.45 p m | 100.2 | " | 4.30 p m | 103.8 | " | 5 p m | 103.8 |
| " | 6.20 p m | 101.8 | " | 6.45 p m | 101. | " | 6.30 p m | 103.9 | " | 7 p m | 103.5 |
| " | 8.20 p m | 101.4 | " | 8.45 p m | 101.3 | " | 8.45 p m | 103. | | | |
| " | 10.20 p m | 101. | " | 11 p m | 101.2 | | | | | | |
| Apr. 7, | 6.20 a m | 99.8 | Ap. 11, | 6 a m | 99.9 | Ap. 14, | 9 a m | 101.3 | Ap. 20, | 9 a m | 101.5 |
| " | 12.20 p m | 99.4 | " | 10.20 a m | 99.8 | " | 12.30 p m | 101.3 | " | 3 p m | 102.1 |
| " | 4.20 p m | 99. | " | 2 p m | 90.8 | " | 4.30 p m | 101.9 | " | 7 p m | 103. |
| Ap. 8, | 9 a m | 99. | " | 4.20 p m | 100. | " | 6.30 p m | 101.9 | | | |
| " | 1.30 p m | 99.1 | " | 10.20 p m | 100. | " | 9.30 p m | 101.5 | | | |
| " | 6.15 p m | 100. | | | | Ap. 15, | 9 a m | 100. | | | |
| | | | | | | " | 2 p m | 101.4 | | | |
| | | | | | | " | 5.40 p m | 103. | | | |

### TABLE No. II.

### *Experiments with Mallein.*

| | I. | | | II. | | | |
|---|---|---|---|---|---|---|---|
| | | | Glandered. | | | Glandered. Reinjected (2). | Healthy. |
| | | | No. 7. | | | No. 7. | No. 8. |
| | Date. | Time. | Temp. | Date. | Time. | Temp. | Temp. |
| | Dec. 11, | 7 a m | 100.2 | Jan. 26, | 4.30 p m | 100.6 | 100.4 |
| | " | 10 a m | 100.7 | " 27, | 8 a m | 99.8 | 98.2 |
| Before | " | 1 p m | 100.8 | " | 10 a m | 100.2 | 99.2 |
| Injection. | " | 4 p m | 101. | " | 1 p m | 100.2 | 99.4 |
| | " | 7 p m | 100.8 | " | 4.30 p m | 100.2 | 99.8 |
| | " | 10 p m | 100.6 | | | | |

| | Date | Time | Inj. 2 cc. | Date | Time | Inj. 1 cc. | Inj. 1 cc. |
|---|---|---|---|---|---|---|---|
| | Dec. 12, | 7 a m | 100.6 | Jan. 28, | 8.30 a m | 100.2 | 99.4 |
| | " | 10 a m | 102.4 | " | 10.15 a m | 101.2 | 99.8 |
| | " | 11.30 a m | 103.8 | " | 11.15 a m | 102.4 | 101.4 |
| When | " | 12.30 p m | 104.2 | " | 1 p m | 102.2 | 101.8 |
| Injected. | " | 1.30 p m | 104.6 | " | 4 p m | 103.5 | 104.5 |
| | " | 2.30 p m | 105.2 | " | 7 p m | 103. | 103.2 |
| | " | 4 p m | 105.3 | | | | |
| | " | 5 p m | 105.1 | | | | |
| | " | 9.30 p m | 104.5 | | | | |

| | Date | Time | Very Large Swelling. | Date | Time | Very Large Swelling. | Slight Swelling. |
|---|---|---|---|---|---|---|---|
| | Dec. 13, | 7.30 a m | 103.2 | Jan. 29, | 8 a m | 101.6 | 100. |
| | " | 10 a m | 102.6 | " | 10 a m | 101.6 | 101.2 |
| | " | 1 p m | 102.5 | " | 1 p m | 102.9 | 100.9 |
| | " | 4 p m | 102. | " | 4 p m | 101.6 | 100.8 |
| After | " | 9.30 p m | 101.6 | " | 7 p m | 102.2 | 99.6 |
| Injection. | Dec. 14, | 8 a m | 101.6 | Jan. 30, | 8 a m | 101. | 101.2 |
| | " | 1 p m | 102. | " | 10 a m | 101.6 | 101.5 |
| | " | 5 p m | 101.2 | " | 1 p m | 101.2 | 101.2 |
| | Dec. 15, | 8 a m | 100.2 | " | 4 p m | 101.5 | |
| | | | | Jan. 31, | 8.30 a m | 100. | 100. |
| | | | | Feb. 1, | 9 a m | 100.4 | 100.2 |
| | | | | " 2, | | | 101 |

**III.**

| Date. | Time. | Farcy No. 10. Temp. | Heathy No. 9. Temp. |
|---|---|---|---|
| | | Inj. 1 cc. | 1 cc. |
| Feb. 1, | 9 a m | 99.5 | 99. |
| " | 10.30 a m | 100.2 | 99.8 |
| " | 12 m | 100. | 99.2 |
| " | 3 p m | 101.2 | 100.3 |
| " | 6 p m | 102. | 100.8 |
| " | 9 p m | 103.2 | 101.8 |
| " | 11 p m | 105. | 102.6 |
| | | Large Swelling. | Very Slight Swelling. |
| Feb. 2, | 8 a m | 103.2 | 100.6 |
| " | 10.30 a m | 102.8 | 100.4 |
| " | 1 p m | 102.8 | 100.4 |
| " | 4 p m | 103. | 100.8 |
| " | 8.20 p m | 103. | 101.2 |
| Feb. 3, | 8 a m | 102.2 | 101. |
| " | 10 a m | 102.2 | 101.3 |
| " | 2 p m | 101.6 | |
| " | 5 p m | 101.9 | |

**IV.**

| Date. | Time | Glandered Reinj. (3) No. 7. Temp. | Farcy Reinj. (2) No. 10. Temp. | Heal'y Reinj. (2) No. 8. Temp. | Heal'y Reinj (2) No. 9. Temp. | Heal'y No. 11. Temp. | Heal'y No. 12 Temp |
|---|---|---|---|---|---|---|---|
| Feb. 7, | 9 a m | 100.8 | 100 | 99.4 | 100. | 100.5 | 99.8 |
| " | 1 p m | 101.2 | 100. | 100. | 100.6 | 101.2 | 100.2 |
| " | 5 p m | 101.2 | 100.2 | 99.8 | 100.5 | 101.2 | 99.8 |
| | | Inj. 1 cc | Inj. 1 cc. | Inj. 1 cc. | Inj. 1 cc. | Inj. 1 cc. | Inj. 1 cc |
| Feb. 8, | 8 a m | 99.9 | 99.8 | 99.6 | 100. | 101. | 100. |
| " | 10 a m | 100.8 | 100.6 | 100. | 100.5 | 101.2 | 99.8 |
| " | 12 m | 101.4 | 100.2 | 100. | 100.2 | 100.8 | 100.2 |
| " | 3 p m | 103.3 | 102.6 | 100.6 | 100.2 | 100.4 | 100. |
| " | 6 p m | 103.6 | 104. | 100.4 | 101. | 100.8 | 100. |
| " | 9 p m | 104. | 103.4 | 100. | 100.8 | 101.2 | 100.2 |
| " | 12 mid | 103. | 103.2 | 99.6 | 101.8 | 101.2 | 100.4 |
| | | Large Swell'g. | Large Swell'g. | No Swell'g. | No Swell'g. | No Swell'g | No Swell'g |
| Feb. 9, | 8 a m | 102.2 | 100.6 | 100.2 | 99.8 | 100.5 | 100.1 |
| " | 1 p m | 103. | 101.2 | 99.8 | 100.9 | 100.8 | 99.8 |
| " | 5 p m | 102.8 | 102.2 | 100.6 | 100.8 | 101.2 | 100. |
| Feb. 10, | 8 a m | 101. | 101. | 98.4 | 101.2 | 101.2 | 100. |
| " | 1 p m | 101.3 | 101.2 | 99.8 | 100.8 | 101.3 | 99.8 |
| " | 6 p m | 101.4 | 101. | 100.4 | 100.8 | 101.2 | 100.2 |

**V.**

| Date. | Time. | Farcy Reinj.(3) No. 10. Temp. | Gland'd No. 14. Temp. | Heal'y Reinj.(3) No. 8. Temp. | Heal'y No. 13. Temp. | Heal'y No. 15.* Temp. |
|---|---|---|---|---|---|---|
| Feb. 12, | 9 a m | 100. | 102.4 | 100. | 100.6 | 101.8 |
| " | 1 p m | 101. | 101.6 | 100.5 | 101. | 102. |
| " | 5 p m | 101.6 | 101.8 | 99.8 | 100.6 | 101.6 |
| | | Inj. 1 cc. | Inj. 1 cc. | Inj. 3 cc. | Inj. 1 cc. | Inj. 1 cc. |
| Feb. 13, | 8 a m | 100.2 | 101.3 | 100.4 | 100.4 | 101.6 |
| " | 10 a m | 100.6 | 101.6 | 99. | 100.6 | 100.8 |
| " | 12 m | 100.6 | 101.6 | 100.4 | 101. | 100.8 |
| " | 2 p m | 101.8 | 102.2 | 100.5 | 100.8 | 101.8 |
| " | 5 p m | 103.2 | 104.8 | 100.8 | 101.6 | 102.8 |
| " | 10 p m | 103.8 | 105. | 99.4 | 101.2 | 101.5 |
| " | 12 mid. | 104.4 | 105. | 100.4 | 102. | 101.2 |
| | | Large Swell'g. | Very Large Swell'g. | No Swell'g. | No Swell'g. | No Swell'g. |
| Feb. 14, | 8 a m | 101.4 | 103.4 | 100.4 | 100. | 101. |
| " | 1 p m | 101.8 | 104. | 99. | 100.6 | 100.6 |
| " | 5 p m | 102. | 103.2 | 100.8 | 100.6 | 101.6 |
| Feb. 15, | 8 a m | 100.6 | 103.2 | 100.6 | 100.2 | 101.6 |
| " | 1 p m | 100. | 102.6 | 99. | 100.6 | 100.6 |
| " | 5 p m | 100.6 | 103.8 | 99.9 | 100.9 | 101.2 |
| Feb. 16, | 8 a m | 100. | 102.8 | 99.9 | 100.6 | 100.9 |
| " | 12 m | 100. | 102.8 | 100. | 100.2 | 101. |
| " | 5 p m | 100. | 102.4 | 99.8 | 99.8 | |

**VI.**

| Date. | Time. | Gland'd Reinj.(2) No. 14. Temp. | Heal'y Reinj.(2) No. 13. Temp. | Heal'y No. 16. Temp |
|---|---|---|---|---|
| Feb. 17, | 9 a m | 103.6 | 99.6 | 98.8 |
| " | 1 p m | 103. | 99.8 | 99.5 |
| " | 5 p m | 103. | 100.6 | 100. |
| | | Inj. 1 cc. | Inj. 1 cc. | Inj. 1 cc. |
| Feb. 18, | 8 a m | 101.5 | 99. | 98.6 |
| " | 10 a m | 101.2 | 99.2 | 98.9 |
| " | 12 m | 101. | 98.8 | 98.9 |
| " | 2 p m | 101. | 99.6 | 98.8 |
| " | 4 p m | 102.2 | 100.2 | 99 4 |
| " | 7 p m | 102.2 | 100.6 | 99. |
| " | 10 p m | 101.8 | 100.2 | 98.8 |
| | | Large Swell'g. | No Swell'g. | No Swell'g |
| Feb. 19, | 8 a m | 101.5 | 99.8 | 99.6 |
| " | 1 p m | 101.5 | 100. | 100.2 |
| " | 5 p m | 102.4 | 99.8 | 99.8 |
| Feb. 20, | 8 a m | 103.6 | 100. | 99.4 |
| " | 1 p m | 103.2 | | 104.4 |
| " | 5 p m | 103.8 | | |
| Feb. 21, | 8 a m | 102.6 | | |
| " | 5 p m | 102.2 | | |
| Feb. 22, | 8 a m | 103.8 | | |

**VII.**

| | Date. | Time. | Gland'd Reinj. (4) No. 7. Temp. | Gland'd Reinj.(3) No. 14. Temp. | Heal'y Reinj.(2) No. 16. Temp. | Heal'y No. 17. Temp. |
|---|---|---|---|---|---|---|
| | Feb. 22, | 9 a m | 98.8 | 103.8 | 98.5 | 98.4 |
| | " | 5 p m | 99.6 | 103.8 | 100. | 100.5 |
| Before | Feb. 23, | 8 a m | 99.4 | 102.8 | 99.2 | 100.2 |
| Injection. | " | 1 p m | 99.8 | 102.8 | 99.8 | 100. |
| | " | 5 p m | 100.1 | 103.2 | 99.8 | 101.4 |
| | Feb. 24, | 8 a m | 100. | 102.2 | 99.5 | 100. |
| | | | Inj. 1 cc. | Inj. 1 cc. | Inj. 1 cc. | Inj. 1 cc. |
| | Feb. 24, | 10 a m | 100.2 | 102.2 | 99.4 | 104.4 |
| | " | 12 m | 100.2 | 102.2 | 99.8 | 99.5 |
| | " | 2 p m | 100.4 | 102. | 100. | 100. |
| When | " | 4 p m | 100.4 | 102.4 | 100.4 | 101.1 |
| Injected. | " | 6 p m | 101.2 | 102.4 | 99.6 | 101.2 |
| | " | 8 p m | 100.2 | 103.2 | 98.5 | 100.8 |
| | " | 10.30 p m | 102.5 | 102.8 | 99.7 | 101.1 |

**VIII.**

| Date. | Time. | Gland'd No. 19. Temp. | Heal'y No. 18. Temp. |
|---|---|---|---|
| Mar. 30, | 1 p m | 101.4 | 99.6 |
| " | 6 p m | 101.4 | 101. |
| | | Inj. 1 cc. | Inj. 1 cc |
| Mar. 31, | 6 a m | 101.6 | 99.4 |
| " | 8 a m | 101.2 | 99. |
| " | 10 a m | 101.4 | 99.3 |
| " | 12 m | 102. | 99 1 |
| " | 2 p m | 102.9 | 100. |
| " | 4 p m | 102.9 | 100.2 |
| " | 6 p m | 103.4 | 100.4 |
| " | 8 p m | 104. | 100.9 |
| " | 10 p m | 103.8 | 100.2 |

* Mare in heat.

| | | | Large Swell'g. | Large Swell'g. | No Swell'g. | No Swell'g. | | | Very Large Swell'g. | No Swell'g |
|---|---|---|---|---|---|---|---|---|---|---|
| | Feb. 25, | 1 a m | 102.5 | 102.6 | 99.8 | 101.5 | Apr. 1, | 7.30 a m | 103.7 | 99.3 |
| | " | 8 a m | 101. | 102.7 | 99.5 | 99.8 | " | 10 a m | 103. | 99.8 |
| | " | 1 p m | 101.6 | 103.2 | 99.4 | 100.4 | " | 1 p m | 103. | 99.1 |
| After | " | 5 p m | 102.6 | 103.2 | 99.8 | 100. | " | 5 p m | 103. | 99.8 |
| Injection. | Feb. 26, | 8 a m | 101.6 | 102.8 | 99.8 | 100.4 | Apr. 2, | 7 a m | 101.4 | 99.2 |
| | " | 1 p m | 102. | 103.6 | 100.2 | 100.6 | | | | |
| | " | 5 p m | 101.4 | 103.2 | 99.4 | 100.6 | | | | |

### IX.

| | | Glandered Reinj. (2) No. 19. | Healthy Reinj. (2) No. 18. |
|---|---|---|---|
| Date. | Time. | Temp. | Temp. |
| Apr. 4, | 8 a m | 101.1 | 99. |
| " | 1 p m | 100.8 | 100.8 |
| " | 1.30 p m | | 100.3 |
| " | 5 p m | 102.2 | 99. |
| Apr. 5, | 8 a m | 101.5 | 98.8 |
| " | 1 p m | 101. | 99. |
| " | 5 p m | 101.8 | 100.4 |
| | | Inj. 1 cc. | Inj. 1 cc. |
| Apr. 6, | 6 a m | 102.4 | 99.4 |
| " | 8 a m | 102.6 | 99. |
| " | 10 a m | 102.7 | 99.2 |
| " | 12 m | 102.1 | 99.6 |
| " | 2 p m | 102.8 | 99.6 |
| " | 4 p m | 102.8 | 99.6 |
| " | 6 p m | 102.4 | 100.4 |
| " | 8 p m | 102.4 | 100.5 |
| " | 10 p m | 103.4 | 100.4 |
| | | Slight Swelling. | No Swelling. |
| Apr. 7, | 8 a m | 102. | 99.2 |
| " | 12 m | 101. | 99. |

### X.

| | | Glandered Reinj. (3) No. 19. | Healthy. Reinj. (2) No. 11. |
|---|---|---|---|
| Date. | Time. | Temp. | Temp. |
| Apr. 25, | 9 a m | 101. | 100.2 |
| " | 1 p m | 101.3 | 100.5 |
| " | 5 p m | 102.4 | 101. |
| | | Inj. 1 cc. | Inj. 1 cc. |
| Apr. 26, | 6 a m | 103.6 | 99.4 |
| " | 8 a m | 103. | 100.2 |
| " | 10 a m | 102.8 | 101.2 |
| " | 12 m | 102.4 | 101.8 |
| " | 2 p m | 102. | 101.8 |
| " | 4 p m | 102.2 | 101.4 |
| " | 6 p m | 102.8 | 100.5 |
| " | 8 p m | 102. | 100. |
| " | 10 p m | 102.8 | 100. |
| | | Slight Swelling. | No Swelling. |
| Apr. 27, | 7.30 a m | 101.6 | 100. |
| " | 10 a m | 101.4 | 100.6 |
| " | 1 p m | 102.4 | 100.6 |

### XI.

| | | Farcy No. 20. | Healthy Reinj. (2) No. 15. |
|---|---|---|---|
| Date. | Time. | Temp. | Temp. |
| May 23, | 8 a m | 100.4 | 101.* |
| " | 12 m | 100.2 | 100.8 |
| " | 5 p m | 99.5 | 99.5 |
| | | Inj. 1 cc. | Inj. 1 cc. |
| May 24, | 6 a m | 100. | 101.2 |
| " | 8 a m | 100.2 | 100.6 |
| " | 10 a m | 100.2 | 100.6 |
| " | 12 m | 100.6 | 100.8 |
| " | 2 p m | 102.4 | 101.2 |
| " | 4 p m | 103.4 | 102. |
| " | 6 p m | 103.8 | 101.8 |
| " | 8 p m | 104.8 | 102.4 |
| " | 10 p m | 104.9 | 101.8 |

### XII.

| | | Farcy Reinj. (2) No. 20. | Farcy No. 21. | Healthy Reinj. (3) No. 15. |
|---|---|---|---|---|
| Date. | Time. | Temp. | Temp. | Temp. |
| May 27, | 12 m | 101.2 | 100.8 | 100.4 |
| " | 5 p m | 102.4 | 100.4 | 100.4 |
| | | Inj. 1 cc. | Inj. 1 cc. | Inj. 1 cc. |
| May 28, | 6 a m | 101.6 | 100.6 | 100.4 |
| " | 8 a m | 101.4 | 100. | 100.2 |
| " | 10 a m | 102. | 100. | 100.2 |
| " | 12 m | 101.4 | 100. | 100.6 |
| " | 2 p m | 101.6 | 101.5 | 100.8 |
| " | 4 p m | 101.8 | 102.6 | 101. |
| " | 6 p m | 101.8 | 104.3 | 101.7 |
| " | 8 p m | 101.8 | 104.4 | 101.5 |
| " | 10 p m | 101.7 | 104.4 | 101.6 |

*Mare in heat.

| | | Large Swelling. | Slight Swelling. | | | Very Large Swelling. | Large Swelling. | Swelling. |
|---|---|---|---|---|---|---|---|---|
| May 25, | 6 a m | 103. | 100.8 | May 29, | 6 a m | 102.4 | 101.6 | 101. |
| " | 9 a m | 102.8 | 100.4 | " | 12 m | 102.9 | 101.4 | 101.2 |
| " | 12 m | 102. | 100.8 | " | 5 p m | 103.2 | 102.6 | 101. |
| " | 3 p m | 102.2 | 101.2 | May 30, | 6 a m | 101.8 | 100.8 | 101. |
| " | 6 p m | 101.8 | 100.5 | " | 5 p m | 102.6 | 102.4 | |
| May 26, | 6 a m | 102.6 | 100.4 | | | | | |

### TABLE No. III.

### *Experiments with 0.5 cc. Mallein.*

**I.**

| | Date. | Time. | Glandered No. 25. Temp. | Glandered No. 26. Temp. | Healthy No. 27. Temp. |
|---|---|---|---|---|---|
| | July 10, | 8 a m | 101.2 | | |
| Before | " | 5 p m | 101 4 | | |
| Injection. | July 11, | 8 a m | 101.4 | 102. | 99.6 |
| | " | 1 p m | 101.4 | 101.8 | 99.8 |
| | " | 6 p m | 101.6 | 101.8 | 99.4 |
| | | | Inj. 0.5 cc. | Injec. 0.5 cc. | Inj. 0.5 cc. |
| | July 12, | 6 a m | 101.2 | 101.2 | 99.4 |
| | " | 8 a m | 101.8 | 100.6 | 99.2 |
| When | " | 10 a m | 101.4 | 101.2 | 99.8 |
| Injected. | " | 12 m | 101.6 | 101.8 | 99.2 |
| | " | 2 p m | 103. | 103.4 | 99.4 |
| | " | 4 p m | 103.4 | 104.2 | |
| | " | 6 p m | 103.2 | 103.8 | 99. |
| | " | 8 p m | 104. | 103.2 | 99.8 |
| | " | 10 p m | 103.8 | 102.5 | 101. |
| | | | Swelling. | Large Swelling. | Swelling Barely Perceptible. |
| | July 13, | 6 a m | 101.8 | 100. | 100.2 |
| | " | 12 m | | 101.6 | 100.4 |
| After | " | 6 p m | | 102.8 | 100.6 |
| Injection. | July 14, | 8 a m | | 100.7 | 99.8 |
| | " | 4 p m | | 100.2 | 100. |
| | July 15, | 7 p m | | 99.8 | 100.2 |

**II.**

| Date. | Time | Farcy Reinj. (8) No. 20. Temp. | Farcy Reinj. (6) No. 21. Temp. | Gland'd No. 34. Temp. | Gland'd No. 35. Temp. | Heal'y No. 33. Temp. | Heal'y No. 36. Temp. | Heal'y No. 37. Temp. | Heal'y. No. 38. Temp. |
|---|---|---|---|---|---|---|---|---|---|
| Aug. 26, | 6 a m | 99.4 | 99.9 | 102.8 | 101.8 | 99.2 | 99.6 | 99.8 | 100. |
| " | 12 m | 100.4 | 100.5 | 103. | 102.8 | 99.9 | 100.4 | 100.4 | 100. |
| " | 6 p m | 100. | 99.6 | 102.8 | 104.3 | 99.9 | 100 8 | 100. | 100.2 |

| | | | | | | | | | |
|---|---|---|---|---|---|---|---|---|---|
| | | Inj. 0.5 cc. | | Inj. 0.5 cc. | | Inj. 0.5 cc. | | Injec. 0.5 cc. | |
| Aug. 27, | 6 a m | 99.6 | 99.4 | 102. | 101.8 | 99.6 | 100.2 | 99.4 | 100.2 |
| " | 8 a m | 99.8 | 99.7 | 101.8 | 101.6 | 99 1 | 99 5 | 99.1 | 99.7 |
| " | 10 a m | | 100.3 | 101.3 | 101.4 | 99. | 99.8 | 99.9 | 100.3 |
| " | 12 m | 100.8 | 101.3 | 102.1 | 102 2 | 99.4 | 99.9 | 100.4 | 100.6 |
| " | 2 p m | 101.2 | 102.7 | 103. | 103. | 99.8 | 100 6 | 100.2 | 100.9 |
| " | 4 p m | 102.8 | 103.2 | 103.6 | 103.4 | 100.5 | 101. | 100.4 | 102 2 |
| " | 6 p m | 103. | 103 6 | 103.7 | 103.7 | 101.2 | 101.4 | 100.5 | 102.7 |
| " | 8 p m | 103. | 103.6 | 104.1 | 103.6 | 101.1 | 101.4 | 100.4 | 102.4 |
| " | 10 p m | 103. | 101. | 104. | 103.6 | 101. | 101.5 | 100.4 | 101.4 |
| | | | | Large | Large | Slight | Slight | Slight | Slight. |
| | | Swell'g. | Swell'g. | Swell'g. | Swell'g. | Swell'g. | Swell'g. | Swell'g. | Swell'g. |
| Aug. 28, | 6 a m | 101 4 | 101. | 102.4 | 102.4 | 99.9 | 101.2 | 100.4 | 100.2 |
| " | 1 p m | 102.9 | 102.9 | 102.2 | 102. | 99.8 | 100.8 | 100.3 | 99.8 |
| " | 6 p m | 101.6 | 101.6 | 103.1 | 102.4 | 99.8 | 100.8 | 100.2 | 100.2 |
| Aug. 29, | 7 a m | 100. | 99.4 | 100.8 | 100.8 | | | 100.2 | 99.8 |
| " | 1 p m | 100.2 | 99.8 | 102.2 | 102. | | | 100. | 99.8 |
| " | 6 p m | 100.2 | 100.2 | 103. | 102.8 | | | 100.4 | 100.2 |

**III.**

| | | | | | | | | | |
|---|---|---|---|---|---|---|---|---|---|
| | | Farcy | Farcy | Gland'd | Gland'd | Gland'd | Heal'y | Heal'y | Heal'y | Heal'y |
| | | Reinj.(9) | Reinj.(7) | Reinj.(2) | Reinj.(2) | | Reinj.(2) | Reinj.[2] | Reinj.[2] | Reinj[2] |
| | | No. 20. | No. 21. | No. 34. | No. 35. | No. 39. | No. 33. | No. 36. | No. 37. | No. 38. |
| Date. | Time. | Temp. | Temp. | Temp. | Temp. | Temp. | Temp. | Temp. | Temp. | Temp. |
| Aug. 29, | 7 a m | 100. | 99.4 | 100.8 | 100.8 | 102.2 | 99.2 | 100.2 | 100.2 | 99.8 |
| " | 1 p m | 100.2 | 99.8 | 102.2 | 102. | 102.6 | 99.6 | 100. | 100 | 99.8 |
| " | 6 p m | 100.2 | 100.2 | 103. | 102.8 | 102.4 | 99.8 | 101.2 | 100.4 | 100.2 |
| | | Inj. 0.5cc. | Inj. 0.5 cc. | | Inj. 0.5 cc. | | Inj. 0.5 cc. | | Inj. 0.5 cc. | |
| Aug. 30, | 6 a m | 99.4 | 99.2 | 102.4 | 100.6 | 101.8 | 99.2 | 100.4 | 99. | 100. |
| " | 8 a m | 99.8 | 100.8 | 102.7 | 100.5 | 101.5 | 99.2 | 100.2 | 100.2 | 100. |
| " | 10 a m | 100.4 | 101.4 | 102.5 | 100.5 | 102.5 | 99.6 | 100.2 | 100.2 | 100.2 |
| " | 12 m | 101 4 | 102.9 | 102.7 | 101.8 | 103.2 | 100. | 100.7 | 100.6 | 100. |
| " | 2 p m | 101.4 | 104.6 | 103.2 | 101.8 | 104.2 | 100.2 | 101. | 100.8 | 100.6 |
| " | 4 p m | 101. | 104.6 | 103.5 | 103.4 | 104.2 | 99.6 | 100.8 | 100.6 | 101.9 |
| " | 6 p m | 101.4 | 104.1 | 103.4 | 103.5 | 104.2 | 100.8 | 100.4 | 100.4 | 102.4 |
| " | 8 p m | 101.3 | 103.4 | 103.8 | 103. | 103. | 110.8 | 101.3 | 100.6 | 102.8 |
| " | 10 p m | | 103. | 103.2 | 102.8 | 102 8 | 100.8 | 101.4 | 100.6 | 102.4 |
| | | Small | Large | Slight | Large | Large | Slight | Slight | Faint | Small |
| | | Swell'g. | Swell'g. | Swell'g. | Swell'g. | Swell'g. | Swell'g. | Swell'g. | Swell'g. | Swell'g. |
| Aug. 31, | 6 a m | 99.8 | 100.6 | 102. | 101.6 | 101.6 | 100. | 101.2 | 100.2 | 100.2 |
| " | 1 p m | 100.7 | 100.4 | 102.4 | 101.6 | 102.6 | 100.4 | 100.8 | 99.8 | 100.7 |
| " | 6 p m | 100.4 | 101.8 | 101.8 | 101.6 | 102.4 | 100.8 | 100.6 | 100.6 | 100.6 |

### TABLE No. IV.

### *Experiments with Albumose.*

**I.**

| | | | | | | | |
|---|---|---|---|---|---|---|---|
| | | | Farcy | Farcy | Glandered | Healthy | Healthy |
| | | | Reinj. [5] | Reinj. [4] | Reinj. [3] | Reinj. [5] | Reinj. [3] |
| | | | No. 20. | No. 23. | No. 21. | No. 11. | No. 17. |
| | Date. | Time. | Temp. | Temp. | Temp. | Temp. | Temp. |
| Before | July 11, | 8 a m | 101. | 100.2 | 100.2 | 99.2 | 99. |
| Injection. | " | 1 p m | 101.2 | 100.6 | 101. | 100.4 | 100.7 |
| | " | 6 p m | 100.6 | 100.2 | 100.6 | 100.4 | 100.8 |

| | | | | | | | |
|---|---|---|---|---|---|---|---|
| | | | .005 grm. Alb. | .005 grm. Alb. | | .005 grm. Alb. | |
| | July 12, | 6 a m | 100. | 96.4 | 99.5 | 100.4 | 100. |
| | " | 8 a m | 100.8 | 99.9 | 100.6 | 100.3 | 100.2 |
| | " | 10 a m | 101. | 100. | 100.8 | 100.8 | 101. |
| When | " | 12 m | 102. | 101. | 102.6 | 101.4 | 101. |
| Injected. | " | 2 p m | 104. | 103.4 | 104.2 | 103. | 100.9 |
| | " | 4 p m | 104.4 | 104.7 | 104.6 | 103.3 | 101. |
| | " | 6 p m | 104.5 | 104.5 | 104.8 | 102.8 | 101.2 |
| | " | 8 p m | 104.2 | 105.8 | 105.2 | 102.8 | 102. |
| | " | 10 p m | 105. | 105 6 | 104.6 | 101.8 | 101.5 |
| | | | Swelling. | Swelling. | Swelling. | Slight Swelling. | Slight Swelling. |
| | July 13, | 6 a m | 103.6 | 104. | 104. | 100. | 99.2 |
| | " | 12 m | 103.8 | 105. | 103.6 | 100.8 | 101. |
| After | " | 6 p m | 102 5 | 104.4 | 103.6 | 100.2 | 101.4 |
| Injection. | July 14, | 8 a m | 100.8 | 101.4 | 102.3 | 100.2 | 99.8 |
| | " | 4 p m | 100.6 | 102. | 102.4 | 100.6 | 100.8 |
| | July 15, | 7 a m | 100.2 | 100.5 | 100.6 | 100.8 | 100. |

**II.**

| | | Farcy Reinj. (6) | Farcy Reinj. (5) | Glandered Reinj. (4) | Glandered Reinj. (2) | Healthy Reinj. (2) | Healthy |
|---|---|---|---|---|---|---|---|
| | | No. 20. | No. 21. | No. 23. | No. 26. | No. 24. | No. 30. |
| Date. | Time. | Temp. | Temp. | Temp. | Temp. | Temp. | Temp. |
| July 21, | 6 a m | 99.4 | 99.5 | 100.6 | 99. | 99.8 | 99. |
| " | 12 m | 99. | 100.8 | 101.5 | 100.4 | 99.6 | 100. |
| " | 5 p m | 98.8 | 100.6 | 100.5 | 102. | 101. | 99.5 |
| | | .002 grm. Alb. | | .002 grm. Alb. | | .002 grm. Alb. | |
| July 22, | 6 a m | 99.8 | 100. | 100.8 | 100. | 99.2 | 99.5 |
| " | 8 a m | 101.4 | 99.2 | 100.8 | 100. | 99.6 | 99. |
| " | 10 a m | 102.6 | 100.8 | 102. | 100.8 | 100. | 99.4 |
| " | 12 m | 101.8 | 101. | 1 02. | 100.5 | 99.8 | 100.2 |
| " | 2 p m | 104.2 | 102.2 | 102.6 | 100.8 | 99.8 | 101. |
| " | 4 p m | 104.2 | 102.2 | 103. | 103.2 | 100. | 100.5 |
| " | 6 p m | 104. | 102.8 | 103.6 | 103. | 99.8 | 100.6 |
| | 10 p m | 104.2 | 102.5 | 104. | 103.8 | 99.4 | 102.6 |
| | | Small Swelling. | Small Swelling. | Large Swelling. | Large Swelling. | No Swelling. | Slight Swelling. |
| July 23, | 6 a m | 100.8 | 101.6 | 102.8 | 101.8 | 100.1 | 99.5 |

**III.**

| | | Farcy Reinj. (7) | Glandered | Healthy | Healthy Reinj. (2) |
|---|---|---|---|---|---|
| | | No. 20. | No. 31. | No. 29. | No. 30. |
| Date. | Time. | Temp. | Temp. | Temp. | Temp. |
| July 26, | 6 a m | 101. | 101.2 | 99.5 | 100.5 |
| " | 12 m | 100.2 | 103. | 101.5 | 100.6 |
| " | 5 p m | 100. | 101.8 | 101.3 | 101.2 |
| | | .002 grm. Alb. | .002 grm. Alb. | .002 grm. Alb. | .002 grm. Alb. |
| July 27, | 6 a m | 100.5 | 100.2 | 101.6 | 100.6 |
| " | 10 a m | 100.6 | 101.4 | 102 8 | 100.8 |
| " | 12 m | 100.8 | 102. | | 99.8 |
| " | 2 p m | 101.4 | 103. | 103. | 101. |
| " | 4 p m | 101. | 104.8 | 102.8 | 101.5 |
| " | 6 p m | 101.5 | 104.2 | 102.4 | 101.4 |
| " | 10 p m | 102. | 103.8 | 101.5 | 101.7 |
| | | Small Swelling. | Large Swelling. | No Swelling. | Slight Swelling. |
| July 28, | 6 a m | 101.2 | 101.8 | 100.2 | 100. |
| ' | 12 m | 102. | 101.7 | 101.2 | 100.2 |

